# Dose reduction in CT urography and vasculature phantom studies using model‐based iterative reconstruction

**DOI:** 10.1120/jacmp.v17i6.6184

**Published:** 2016-11-08

**Authors:** Leland Page, Wei Wei, Vikas Kundra, X. John Rong

**Affiliations:** ^1^ Department of Imaging Physics The University of Texas MD Anderson Cancer Center Houston TX USA; ^2^ Department of Biostatistics The University of Texas MD Anderson Cancer Center Houston TX USA; ^3^ Department of Diagnostic Radiology The University of Texas MD Anderson Cancer Center Houston TX USA; ^4^ Department of Cancer Systems Imaging The University of Texas MD Anderson Cancer Center Houston TX USA

**Keywords:** CT, urography, vasculature, iterative reconstruction, dose

## Abstract

To evaluate the feasibility of radiation dose reduction using model‐based iterative reconstruction (MBIR) for evaluating the ureters and vasculature in a phantom, a tissue‐equivalent CT dose phantom was scanned using a 64‐channel CT scanner. Tubes of varying diameters filled with different dilutions of a contrast agent, simulating ureters or vessels, were inserted into the center of the phantom. Each combination was scanned using an existing renal protocol at 140 kVp or 120 kVp, yielding a display volumetric CT dose index (${\text{CTDI}}_{\text{vol}}$) of 24 mGy. The scans were repeated using reduced scan techniques to achieve lower radiation doses down to 0.8 mGy. The images were reconstructed using filtered back‐projection (FBP) and model‐based iterative reconstruction (MBIR). The noise and contrast‐to‐noise ratio (CNR) was measured for each contrast object. Comparisons between the two reconstruction methods at different dose levels were evaluated using a factorial design. At each ${\text{CTDI}}_{\text{vol}}$ the measured image noise was lower using MBIR compared to FBP (p<0.0001). At low doses, the percent change in measured image noise between FBP and MBIR was larger. For the 12 mm object simulating a ureter or large vessel with an HU of 600, the measured CNR using MBIR at a ${\text{CTDI}}_{\text{vol}}$ of 1.7 mGy was greater than the CNR of FBP at a ${\text{CTID}}_{\text{vol}}$ of 24 mGy (p<0.0001). For the 5 mm object simulating a medium‐sized vessel with a HU of 250, the measured CNR using MBIR at a ${\text{CTDI}}_{\text{vol}}$ of 1.7 mGy was equivalent to that of FBP at a ${\text{CTDI}}_{\text{vol}}$ of 24 mGy. For the 2 mm, 100 HU object simulating a small vessel, the measured CNR using MBIR at a ${\text{CTDI}}_{\text{vol}}$ of 1.7 mGy was equivalent to that of FBP at a ${\text{CTDI}}_{\text{vol}}$ of 24 mGy. Low‐dose (3.6 mGy) CT imaging of vasculature and ureter phantoms using MBIR results in similar noise and CNR compared to FBP at approximately one‐sixth the dose. This suggests that, using MBIR, a one milliSievert exam of the ureters and vasculature may be clinically possible whilst still maintaining adequate image quality

PACS number(s): 87.57.‐s, 87.57.Q‐, 87.57.C‐, 87.57.nf, 87.57.cj, 87.57.cm

## I. INTRODUCTION

Over the past ten years, there have been many advances in computed tomography (CT) technology. These advancements continue to make CT a valuable diagnostic tool. There is, however, a growing concern over the potential risks for developing radiation‐induced cancers from CT scans.^(1)^ In some cases, patients undergo multiple CT examinations — for example, to diagnose and monitor diseases such as urethral cancers — resulting in high accumulated radiation dose.

CT is an excellent method for evaluating pathology, but achieving adequate image quality for proper diagnosis can be problematic if the dose is too low.^2^, ^3^

CT urography is useful for evaluation of calculi, renal masses, and urothelial tumors. It has been shown that CT urography outperforms ultrasound, excretory urography, and radiography in the evaluation of renal parenchymal masses and urinary tract calculi.^(4)^ Radiation doses associated with CT urography have been measured to be anywhere from 1.5–2 times the dose of standard urography^(5)^ with effective doses as high as 35 mSv reported for some CT urography studies.^(6)^

Due to the potential risks associated with these scans, methods to reduce the amount of radiation dose delivered to the patient while still achieving adequate diagnostic image quality need to be explored. This is especially the case for younger patients where there is a need for scanning using low‐dose techniques due to a larger lifetime attributable risk of radiation‐induced cancers.^(7)^ Many studies use a split‐bolus protocol in patients under the age of 40,^8^, ^9^ acquiring at least 2 phases. A history of urothelial cancer can require life‐time surveillance with multiple CT scans. Both the ureters and vessels are commonly imaged when opacified with contrast with HU considerably greater than organ parenchyma.

Recently, capitalizing on advancements in computing power and reconstruction technology, a model‐based iterative reconstruction (MBIR) technology, called “Veo”, was implemented.^(10)^ Since the early inception of CT, filtered back‐projection (FBP) has been the primary method of CT image reconstruction. FBP is fast and simple, due to assumptions made about the noise in the signal and the geometry of system optics.^(11)^ FBP assumes that the X‐ray focal spot is a point source. It also uses a pencil beam as a model of the path of the X‐ray source to the detector.^(12)^ These assumptions lead to inaccuracies in the reconstructed images and suboptimal spatial resolution. It also assumes that each projection measurement is not influenced by changes in photon statistics or electronic noise. This could propagate and amplify noise in the images, hiding pathology and losing important diagnostic information. MBIR performs multiple iterations from multiple models. These models overcome the limitations of FBP by accounting for the system optics and geometry effects,^(13)^ as well as noise from photon flux and noise inherent to the system. The use of MBIR in CT urography studies has not been explored to address how much the radiation dose can be decreased while still maintaining adequate diagnostic quality in terms of CT number and image noise comparable to higher doses commonly used with FBP.

The diagnosis of CT urography focuses primarily on evaluation of both ureters and vasculature, which are enhanced with contrast agent, and therefore there is a potential for low‐dose patient imaging in CT urography studies. The availability of MBIR provides an opportunity of reducing patient dose while maintaining adequate characteristics of ureters and vasculature in CT urography images. At present, it is unclear at what dose using MBIR can the same image quality be achieved as when using standard FBP reconstruction and technique in CT urography studies. Utilizing new reconstruction models could allow for the evaluation of both ureters and vasculature on more routine exams and could increase the utility of CT urography screening for urothelial carcinomas without the trade‐off of increased accumulative doses to the patient. The purpose of this study is to evaluate the feasibility of radiation dose reduction using MBIR for evaluating the ureters and vasculature in a phantom.

## II. MATERIALS AND METHODS

**Figure 1:**
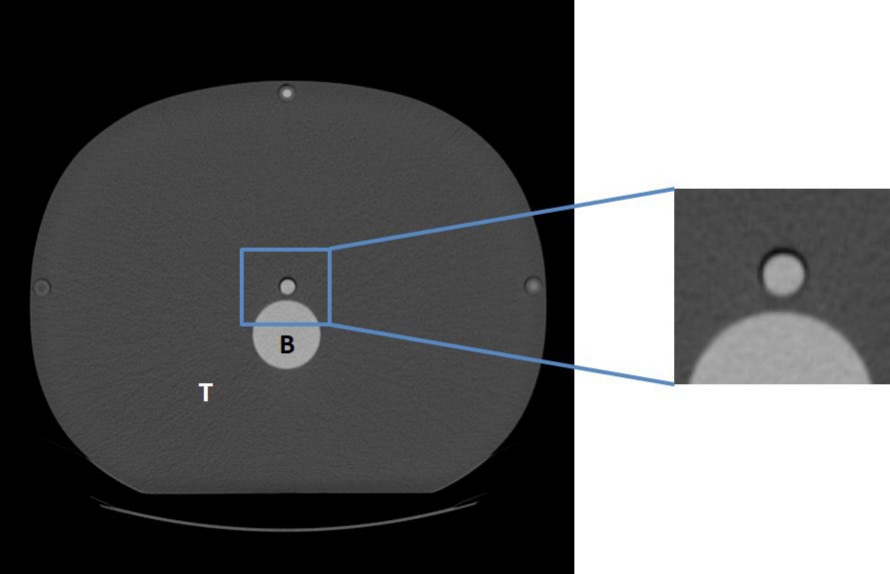
Image of the CIRS phantom with the 12 mm, 600 HU object used to simulate the ureters. The image on the right is a zoomed portion of the phantom containing the contrast object: B=bone‐equivalent; T=tissue‐equivalent.

A large adult abdominal tissue‐equivalent CT dose phantom (CIRS 007TE‐08; CIRS Inc., Norfolk, VA) was used. It contains five through‐holes with a diameter of 1.3 cm made to accommodate CT dose probes. One of the holes is at the center and four are around the perimeter. In order to simulate the anatomy of interest, tubes of varying diameters (12 mm, 5 mm, and 2 mm) were filled with different dilutions of a contrast agent (350 mgI/ml Omnipaque (iohexol); GE Healthcare, Princeton, NJ) and inserted into the center hole of the phantom. To achieve the appropriate Hounsfield units (HU) based on measurements on clinical images of the anatomy of interest, the tubes with the contrast agent were diluted with water at concentrations of 1:8, 1:30, and 1:50. The 12 mm diameter tube with a 1:8 concentration was used to simulate the ureters with CT numbers from 600–800 HU. The 5 mm diameter tube with a 1:30 concentration was used to simulate large vasculature, such as arteries, that have CT numbers from 250–300 HU. The 2 mm diameter tube with a 1:50 concentration was used to represent small vasculature with CT numbers of 100 HU. An image of the phantom with the 12 mm tube is shown in Fig. 1.

Each combination of the tube diameter and concentration was scanned on a 64‐channel CT scanner (GE Discovery 750HD; GE Healthcare, Waukesha, WI). A protocol with the following parameters was used to scan the phantom: 140 kVp, 290 mA, 0.8 s rotation time, a pitch of 0.98, and 2.5 mm image thickness. The scans were repeated using lower mA to achieve lower radiation doses. The volumetric CT dose index (${\text{CTDI}}_{\text{vol}}$) reported on the CT scanner was recorded for each of the scan techniques. The scans were also repeated using 120 kVp and mA stations that would yield a ${\text{CTDI}}_{\text{vol}}$ close to those at 140 kVp. Table 1 shows the mA stations used along with the displayed ${\text{CTDI}}_{\text{vol}}$ for each of them.

The images were reconstructed using FBP and MBIR. The FBP reconstructions were acquired at 0.625 mm slice thickness and reconstructed to 2.5 mm thickness using the standard reconstruction kernel. The MBIR images use 0.625 mm slices and were reformatted to achieve 2.5 mm slices.

**Table 1:** mA stations used to scan the phantom for both 140 kVp and 120 kVp, along with the displayed CTDI vol for each

*kVp*	*mA*	${\text{CTDI}}_{\text{vol}}(\text{mGy})$
140	290	24
	165	13.2
	45	3.6
	20	1.6
	10	0.8
120	415	24
	235	13.2
	65	3.6
	30	1.7
	10	0.7

The image noise was measured by placing a region of interest (ROI) at a standard location in the tissue portion of the CIRS phantom and recording the standard deviation within the ROI. The contrast‐to‐noise ratio (CNR) was calculated by using the following equation:

$$CNR=(ROI_{c}-ROI_{p})/SD_{p}$$

were ${\text{ROI}}_{c}$ is the mean signal of the contrast agent, ${\text{ROI}}_{p}$ is the mean signal of the tissue portion of the CIRS phantom, and ${\text{SD}}_{p}$ is the mean image noise of the tissue portion of the CIRS phantom. The average CNR was calculated over five consecutive slices for each image set. Using the combinations of object size, concentration, and ${\text{CTDI}}_{\text{vol}}$, an N of 444 and 438 were analyzed for 140 and 120 kVp, respectively.

Noise and CNR are two commonly used image quality metrics in CT image evaluation. These two metrics are used in this study because CT urography is a limited‐scope study. It has been focused on patient dose reduction, which is largely affected by CNR of low‐contrast objects.

### A. Statistical analysis

This study used a factorial design to estimate differences between FBP and MBIR with respect to image noise and CNR under different image settings in the phantom study. Four image settings were used: kVp, tube diameter, concentration, and ${\text{CTDI}}_{\text{vol}}$. Cubic root transformation was used prior to statistical modeling and analysis results were back‐transformed to raw scale for reporting and plotting. Linear mixed model was used to assess interaction between factors (i.e., image settings and algorithm) first. Interaction terms that were significant led to analysis at each factor level. For example, comparisons between FBP and MBIR for CNR were carried out by kVp, diameter, and concentration. A ${\text{CTDI}}_{\text{vol}}$ of 24 mGy and FBP was set as the reference level for pairwise comparisons. Dunnett's procedure was used to control overall type I error at five percent. All tests were two‐sided and p‐values of 0.05 or less were considered significant. Statistical analysis was carried out using SAS version 9 (SAS Institute, Cary, NC).

## III. RESULTS

### A. Image noise

**Figure 2:**
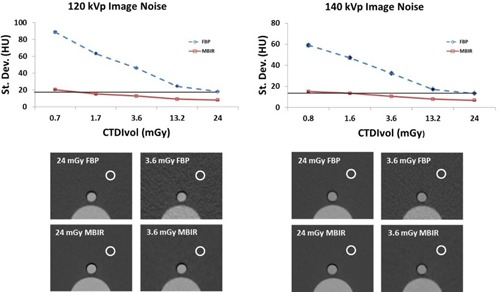
Top: plot comparing image noise of FBP to that of MBIR at 120 kVp (left) and 140 kVp (right). The black horizontal line indicates the average image noise at a ${\text{CTDI}}_{\text{vol}}$ of 24 mGy using FBP. Using MBIR the image noise was less than that of FBP at 24 mGy down to 1.7 mGy at 120 kVp and 3.6 mGy for 140 kVp. Bottom: images of the phantoms at a ${\text{CTDI}}_{\text{vol}}$ of 24 mGy for FBP (top left) and MBIR (bottom left) and 3.6 mGy using FBP (top right) and MBIR (bottom right) are shown below each plot. The circle demonstrates the approximate location where the noise measurements were made in the phantom.

The measured image noise for all of the dose levels using MBIR was significantly lower than FBP (p<0.0001, Fig. 2). At low doses, there is a much larger difference in image noise between MBIR and FBP. At 140 kVp, the mean image noise using MBIR was less than the $24\;\text{mGy}\;{\text{CTDI}}_{\text{vol}}$ FBP at doses down to 3.6 mGy (p<0.0001). At 120 kVp, the mean image noise using MBIR was less than the $24\;\text{mGy}{\text{CTDI}}_{\text{vol}}$ FBP at doses down to 1.7 mGy (p<0.0001).

### B. Contrast‐to‐noise ratio

**Figure 3:**
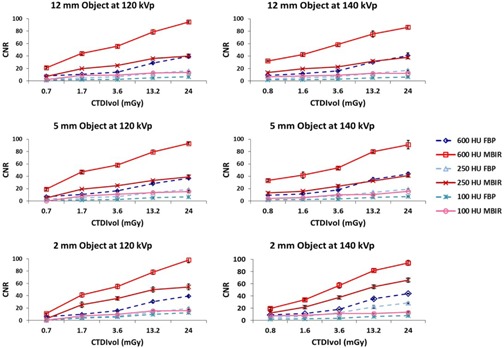
Plots comparing the CNR of MBIR to that of FBP across multiple sizes and Hounsfield units.

The CNR using MBIR at reduced doses (${\text{CTDI}}_{\text{vol}}$ as low as 1.7 mGy) was greater than or equivalent to that using FBP with a ${\text{CTDI}}_{\text{vol}}$ of 24 mGy for the majority of the concentrations and object sizes at both 120 kVp and 140 kVp (Fig. 3).

**Figure 4:**
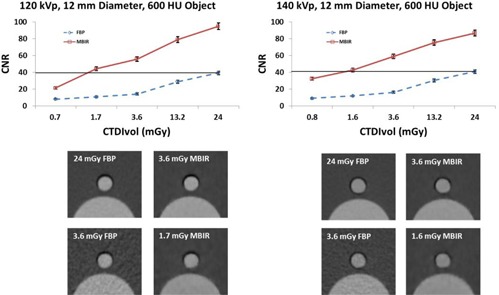
Top: CNR of a 12 mm diameter object with a CT number of approximately 600 HU used to simulate ureters. The line in the plot shows that at 120 kVp the CNR using MBIR at a ${\text{CTDI}}_{\text{vol}}$ of 1.7 mGy was greater than or equal to the CNR using FBP at a ${\text{CTDI}}_{\text{vol}}$ of 24 mGy. For 140 kVp the CNR using MBIR at a ${\text{CTDI}}_{\text{vol}}$ of 1.6 mGy was greater than or equal to the CNR using FBP at a ${\text{CTDI}}_{\text{vol}}$ of 24 mGy. Bottom: images of the phantom at a ${\text{CTDI}}_{\text{vol}}$ of 24 mGy for FBP (top left), 3.6 mGy for MBIR (top right) and a ${\text{CTDI}}_{\text{vol}}$ of 3.6 mGy for FBP (bottom left) and a ${\text{CTDI}}_{\text{vol}}$ of 1.7 mGy (120 kVp) and 1.6 mGy (140 kVp) for MBIR (bottom right) are shown below the plots.

**Figure 5:**
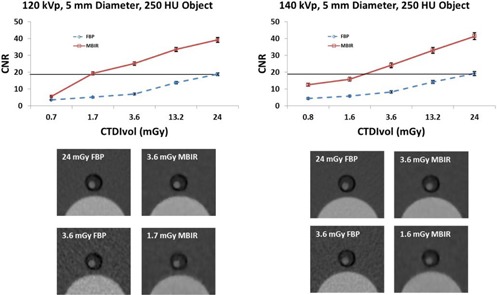
Top: CNR of a 5 mm diameter object with a CT number of approximately 250 HU used to simulate large vasculature. The line in the plot shows that at 120 kVp the CNR using MBIR at a ${\text{CTDI}}_{\text{vol}}$ of 1.7 mGy was greater than or equal to the CNR using FBP at a ${\text{CTDI}}_{\text{vol}}$ of 24 mGy. For 140 kVp the CNR using MBIR at a ${\text{CTDI}}_{\text{vol}}$ of 3.6 mGy was greater than or equal to the CNR using FBP at a ${\text{CTDI}}_{\text{vol}}$ of 24 mGy. Bottom: images of the phantom at a ${\text{CTDI}}_{\text{vol}}$ of 24 mGy for FBP (top left), 3.6 mGy for MBIR (top right) and a ${\text{CTDI}}_{\text{vol}}$ of 3.6 mGy for FBP (bottom left) and a ${\text{CTDI}}_{\text{vol}}$ of 1.7 mGy (120 kVp) and 1.6 mGy (140 kVp) for MBIR (bottom right) are shown below the plots.

**Figure 6:**
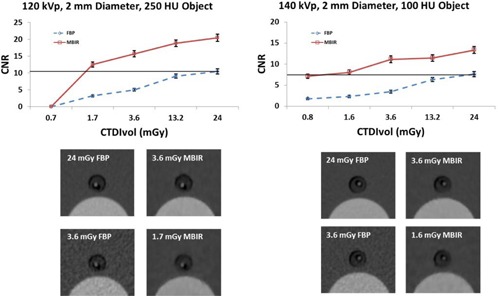
Top: CNR of a 2 mm diameter object with a CT number of approximately 100 HU used to simulate small vasculature. The line in the plot shows that at 120 kVp the CNR using MBIR at a ${\text{CTDI}}_{\text{vol}}$ of 1.7 mGy was greater than or equal to the CNR using FBP at a ${\text{CTDI}}_{\text{vol}}$ of 24 mGy. For 140 kVp the CNR using MBIR at a ${\text{CTDI}}_{\text{vol}}$ of 1.6 mGy was greater than or equal to the CNR using FBP at a ${\text{CTDI}}_{\text{vol}}$ of 24 mGy. Bottom: images of the phantom at a ${\text{CTDI}}_{\text{vol}}$ of 24 mGy for FBP (top left), 3.6 mGy for MBIR (top right) and a ${\text{CTDI}}_{\text{vol}}$ of 3.6 mGy for FBP (bottom left) and a ${\text{CTDI}}_{\text{vol}}$ of 1.7 mGy (120 kVp) and 1.6 mGy (140 kVp) for MBIR (bottom right) are shown below the plots.

The CNR using MBIR for the 12 mm object with a signal of approximately 600 HU, approximating the ureter, was greater than or equal to the CNR of the same object with a ${\text{CTDI}}_{\text{vol}}$ of 24 mGy using FBP down to a ${\text{CTDI}}_{\text{vol}}$ of 1.7 mGy at 120 kVp and 1.6 mGy at 140 kVp (Fig. 4). The CNR using MBIR for the 5 mm object with a signal of approximately 250 HU, simulating a larger vessel, was greater than or equal to the CNR of the same object with a ${\text{CTDI}}_{\text{vol}}$ of 24 mGy using FBP down to a ${\text{CTDI}}_{\text{vol}}$ of 1.7 mGy at 120 kVp and 3.6 mGy at 140 kVp (Fig. 5). The CNR using MBIR for the 2 mm object with a signal of approximately 100 HU, simulating a small vessel, was greater than the CNR of the same object with a ${\text{CTDI}}_{\text{vol}}$ of 24 mGy using FBP down to a ${\text{CTDI}}_{\text{vol}}$ of 1.7 mGy at 120 kVp and 1.6 mGy at 140 kVp (Fig. 6).

## IV. DISCUSSION

To our knowledge, this is the first phantom study addressing the use of MBIR in CT urography or vasculature evaluation. Using MBIR, the dose at which scans are obtained to visualize objects simulating the ureters and vasculature in phantom studies can be reduced by at least a factor of 6 and still maintain adequate image quality in terms of noise and CNR. Conservatively, the findings suggest that a ${\text{CTDI}}_{\text{vol}}$ of 3.6 mGy could be used for CT urography and vasculature studies, leading to an effective dose in the range of 1 mSv. This would aid in reducing the risks associated with the dose received by patients who receive single scans or with a history of urothelial cancer who undergo lifetime surveillance with multiple CT scans.

The measured image noise using MBIR was significantly lower than FBP. At lower doses, there was a much larger difference in image noise, with less noise seen with MBIR. This is important because a reduction in image noise at low doses by using MBIR should allow for better visualization of anatomical structures. A previous study compared MBIR to ASIR and FBP in a torso phantom by using varying noise indices and measuring the mean attenuation and standard deviation of different anatomical areas of the phantom.^(14)^ At the highest noise index, they reported that the measured image noise was 3.5 times lower by using MBIR compared to FBP. A prospective clinical trial of 45 patients who underwent a standard‐dose abdominal CT series immediately followed by an ultralow‐dose abdominal series found the mean image noise reduction by using MBIR to be half that of standard‐dose FBP and three to four times lower than FBP at low doses.^(15)^ These findings support our results. However, our study suggests that even greater reductions by a factor of 6 may be achievable for CT urography and vascular studies.

The CNR by using MBIR at reduced doses down to 3.6 mGy was greater than or equal to FBP at 24 mGy for all object sizes and concentrations at 140 and 120 kVp. For a typical CT urography scan of 30 cm coverage in abdomen–pelvis, the effective dose could be reduced to 1.6 mSv of utilizing MBIR from 10.8 mSv of using FBP. With the larger‐sized objects, the dose reduction was greater than with smaller‐sized objects at CNR equal to FBP at 24 mGy. This was not surprising, given that it is more difficult to visualize finer detailed structures at lower doses. Previous studies of MBIR have been performed on abdominal studies in both adults and children and evaluated image quality in parenchyma of organs such the liver and paraspinal musculature.^15^, ^16^, ^17^ The abdominal studies on adults found a mean dose reduction of 75% by using MBIR. The pediatric study showed an average dose reduction of 45%. Chest studies evaluating lung structure have also been performed,^18^, ^19^ demonstrating a mean dose reduction of 75% by using MBIR. The above studies assessed the dose reduction potential by evaluating the image noise at lower doses. While prior studies have not been performed to specifically evaluate the high‐HU objects such as contrast‐filled ureters or vessels, they support our results. The present study focused on phantoms of contrast‐filled ureters or vessels in the abdomen suggests potential dose reduction approaching 90%.

One of the major drawbacks to using MBIR is the amount of time required to reconstruct the images in a study. Due to the demanding computational requirements, it can take on the order of 30–60 min to reconstruct a series. This could limit the use of MBIR in settings where an immediate diagnosis is needed. However, in a screening environment like CT urography, this should not be a limitation. Another potential drawback of MBIR is that the textures of the images appear unusually smooth or pixelated, which could require some adjustment for clinical interpretation.^16^, ^19^

There are some limitations to this study. First, the data gathered in this study reflect only studies on a standard phantom. This study supports the use of MBIR in CT urogram and vascular studies in future clinical evaluations. However, the results of this study should not be simply expanded to any other CT patient imaging (types of exams and patient populations) where contrast agents are being visualized. Second, this study compared FBP to MBIR. Adaptive statistical iterative reconstruction (ASIR), which is an intermediate dose reduction reconstruction method, was not explored in this study. However, others have reported that ASIR generally results in approximately 30% dose reduction compared to FBP.^(20)^ Third, image noise and CNR cannot represent all aspects of image properties; future research will look into more sophisticated image quality metrics with a broader scope. It is also worth noting, that at lower doses the measured CT numbers using MBIR deviated from those measured using FBP. For this reason, the results of this study should not be expanded to studies where the quantitative analysis is essential to the diagnosis.

## V. CONCLUSIONS

This phantom study demonstrates the potential for performing CT urography and vascular studies using MBIR at reduced doses, approaching those of single mSv exams.

## COPYRIGHT

This work is licensed under a Creative Commons Attribution 3.0 Unported License.
